# Fatty Acid Oxidation and Pro-Resolving Lipid Mediators Are Related to Male Infertility

**DOI:** 10.3390/antiox11010107

**Published:** 2022-01-02

**Authors:** Cinzia Signorini, Elena Moretti, Daria Noto, Lucia Micheli, Rosetta Ponchia, Giulia Collodel

**Affiliations:** 1 Department of Molecular and Developmental Medicine, University of Siena, 53100 Siena, Italy; cinzia.signorini@unisi.it (C.S.); daria.noto@student.unisi.it (D.N.); giulia.collodel@unisi.it (G.C.); 2 Department of Medicine, Surgery and Neurosciences, University of Siena, 53100 Siena, Italy; lucia.micheli@unisi.it; 3 Unit of Medically Assisted Reproduction, Siena University Hospital, 53100 Siena, Italy; ponchia2@student.unisi.it

**Keywords:** DHA, fatty acids, ferritin, F_2_-Isoprostanes, human male infertility, RvD1, seminal inflammation, specialized pro-resolving lipid mediators, TEM

## Abstract

Specialized pro-resolving lipid mediators regulate the resolution of acute inflammation. They are formed by enzymatic oxygenation of polyunsaturated fatty acids and are divided into families including lipoxins, resolvins, protectins, and maresins. Resolvin D1 (RvD1), produced by docosahexaenoic acid, exerts anti-inflammatory and pro-resolving activities. This research aimed to investigate the implication of seminal RvD1 in human infertility. Infertile patients (n° 67) were grouped based on pathological reproductive conditions as idiopathic infertility, varicocele, and leukocytospermia; the fourth group was composed of fertile men (n° 18). Sperm characteristics were evaluated by light microscopy (WHO guidelines) and by transmission electron microscopy (TEM). The seminal levels of RvD1 and F_2_-isoprostane (F_2_-IsoPs) were dosed. In twenty men (6 fertile men, 8 with varicocele, 6 with leukocytospermia) seminal phospholipase A_2_, iron, cholesterol, transferrin, estradiol, ferritin, testosterone, and sperm membrane fatty acids were detected. The results indicated that: (i) RvD1 amount was positively correlated with F_2_-IsoPs and reduced sperm quality; (ii) RvD1 levels were significantly higher in patients with leukocytospermia, varicocele, and idiopathic infertility compared to fertile men; (iii) RvD1 increased along with other markers of oxidative stress and inflammation as fatty acids content and clinical biomarkers. This study suggests a panel of inflammatory markers and lipid mediators for a diagnosis of inflammatory status and a subsequent appropriate therapeutic approach.

## 1. Introduction

As a peculiar evolution of inflammation, which is a humans’ defense mechanism against pathological agents, acute inflammation starts rapidly once biological damage has occurred, resolves in a few days, and is regulated by chemical mediators, such as enzymatic metabolites of arachidonic acid (AA) [1,2,3]. Long-term inflammation and an uncontrolled inflammatory process are believed as critical components in several human diseases [4].

Specialized pro-resolving lipid mediators (SPMs) are reported to be able to regulate the resolution of acute inflammation that should be considered as an active process moved by specific regulatory molecules (i.e., SPMs) [5]. SPMs are formed by enzymatic oxygenation of polyunsaturated fatty acids (PUFAs) and are divided into families including lipoxins, resolvins, protectins, and maresins [6,7]. Interestingly, SPMs do not have direct anti-inflammatory effects by inhibiting or directly blocking this process but can actively reduce neutrophil infiltration into inflamed tissues, enhance bacterial phagocytosis by monocytes and macrophages and simultaneously inhibit inflammatory cytokine production. The switch from the initiation to the resolution phase of acute inflammatory response is crucial for tissue homeostasis, and the failure to resolve early inflammation by specialized pro-resolving mediators leads to chronic inflammation and tissue damage [8].

Resolvins are n-3 eicosapentaenoic acid (EPA) and docosahexaenoic acid (DHA)-derived SPMs [4]. As a component of SPMs, Resolvin D1 (RvD1), produced by DHA, exerts anti-inflammatory and pro-resolving activities in acute inflammation as previously reported [9,10]. In in vivo approach, targets of RvD1-regulated microRNAs have been identified [10]. Therefore, essential PUFAs play key roles in distinct phases (beginning, evolution, and resolution) of acute inflammation, but they are also relevant in semen quality [11,12]. It has been reported that spermatogenesis and fertility are influenced by PUFA content in the sperm plasma membrane [11]. The high content of PUFAs in the sperm plasma membrane, makes them susceptible to lipid peroxidation in presence of oxidative stress (OS) [13]. Male infertility may be due to an inflammation-mediated OS including varicocele, tobacco usage, alcohol, obesity/metabolic syndrome, leukocytospermia, sexually transmitted disease, bacterial prostatitis, and viral infections [14,15,16,17]. Inflammation status may be associated also with idiopathic infertility [18]. Seminal inflammation is a frequent condition, mostly with a chronic course, whose diagnosis may be difficult. Currently, the identification of novel and reliable markers of inflammation in seminal plasma is an open challenge, in particular for male accessory gland inflammation [19]. An inflammatory pathological condition could be diagnosed by reactive oxygen species (ROS) evaluation, but also by the dosage of cytokine levels that have been associated with inflammation regulating spermatogenesis through cell interaction, Toll-like receptors, and production of ROS. Cytokines also affect testosterone production acting at many levels of the pituitary-gonadal axis [20]. Recently Lee et al. [21] have indicated a novel function of RvD1 as a putative IL-6R antagonist suggesting that RvD1-mediated blockade of IL-6 signal transmission may contribute to inhibition of chromosomal instability and tumorigenesis. Other seminal molecules have been suggested as indicators of sperm parameters alterations [22,23].

This paper aimed to provide new insights on the relevance of the axis inflammation-lipid mediators in semen quality to identify a panel of markers able to distinguish different male infertility categories. The definition of a panel of inflammatory markers and lipid mediators could help in defining targeted therapies for infertile men.

## 2. Materials and Methods

### 2.1. Patients

The samples for the study were obtained from 67 infertile selected Italian men (aged 29 to 40 years) attending, from June 2018 to December 2019, the Unit of Medically Assisted Reproduction, at Siena University Hospital for semen analysis. The selected patients had not achieved pregnancy within a period of 1 to 3 years.

We planned to include in the study patients with normal FSH, LH, and testosterone values and a body mass index < 25 kg/m^2^. Exclusion criteria included azoospermia, history of cryptorchidism, occupational chemical exposure, Y chromosome microdeletions, karyotype abnormalities, diabetes, radiotherapy, chemotherapy, use of drugs, alcohol, and dietary supplements.

We also excluded subjects with genitourinary infection, and heavy smoking habits (>10 cigarettes/day).

Varicocele diagnosis was allowed by scrotal eco-color Doppler analysis, leukocytospermia was diagnosed for the presence of leucocytes > 1 × 10^6^/mL in the spermiogram [24].

The patients were grouped in:

(i) idiopathic infertility (n° 23)
(ii) varicocele (n° 21)—where left-sided varicocele was found in 16 cases (grade III in five patients, grade II in seven patients, and grade I in four patients) and right-sided varicocele and bilateral varicocele were diagnosed in two (grade II both) and three (grade I- II), respectively
(iii) leukocytospermia (n° 23)

The fourth group (fertile group) was composed of eighteen fertile men (aged 30–38 years); they fathered at least one child in the last 4 years. Infections, hormonal, and anatomical problems were not present.

To be admitted to the study, participants were informed of the protocol and had to sign a consent for inclusion in the research.

### 2.2. Semen Analysis 

#### Light and Electron Microscopy

Semen samples, obtained by masturbation after 3–5 days of sexual abstinence, were examined following WHO guidelines [24]. Samples were left to liquefy for 30 min at 37 °C. Then, semen volume, pH, sperm concentration, morphology, motility, and vitality were assessed. The evaluation of sperm morphology was performed using the stain-coated testsimplets slides (Origio, Italy). Semen leukocytes were identified using peroxidase stain; leukocytospermia is defined by WHO guidelines [24] as more than 1 million cell/mL.

Transmission electron microscopy analysis, mass spectrometry for F_2_-IsoPs levels detection, and resolvins ELISA assay were carried out for each sample.

For transmission electron microscopy, a part of each semen sample was fixed with cold Karnovsky fixative and stored at 4 °C for 2 h, then centrifuged at 200× *g* for 15 min. Finally, the recovered spermatozoa were rinsed in 0.1 mol/L cacodylate buffer (pH 7.2) for 12 h. Specimens were post-fixed in 1% buffered osmium tetroxide for 1 h at 4 °C and rinsed again in 0.1 mol/L cacodylate buffer. A graded ethanol series was used to dehydrate the samples, that were finally embedded in Epon Araldite resin. The ultra-thin sections, obtained using a Supernova ultramicrotome (Reickert Jung, Vienna, Austria), were collected on copper grids and counterstained with uranyl acetate and lead citrate. Finally, the sections were analyzed and the spermatozoa photographed with a Philips CM12 (Philips Scientifics, Eindhoven, The Netherlands) at the CE.M.E, CNR (Via Madonna del Piano, 10, 50019 Sesto Fiorentino, Italy).

The TEM data were processed using a Bayesian method used in our laboratory for many years [25] which furnishes a fertility index and the percentage of sperm pathologies as necrosis, apoptosis, and immaturity [26]. These sperm pathologies are well defined by peculiar ultrastructural alterations: necrotic sperm show reacted/absent acrosome, altered nuclei with disrupted chromatin texture, damaged plasma membrane, and poor axonemal cytoskeletal structures. Apoptotic sperm have a margination of chromatin, presence of cytoplasmic residues, swollen and unassembled mitochondria. The presence of spherical and elliptical nuclei with uncondensed chromatin, altered acrosomes, and cytoplasmic droplets are markers of immature sperm.

### 2.3. F_2_-Isoprostane (IsoP) Determination

Semen samples were centrifuged at 2000× *g* for 15 min, then the pellet was discarded. At this step, each sample was supplemented with butylated hydroxytoluene (BHT, 100 μM) and was stored at −80 °C.

F_2_-IsoPs are compounds produced by the oxygenation of AA. F_2_-IsoPs are generated as molecules esterified into cell membrane phospholipids before being released in free (unesterified form). Here, total F_2_-IsoPs (esterified plus free F_2_-IsoPs) were analyzed by mass spectrometry. Briefly, at the time of assay, incubation with KOH 1N, 45 °C, 45 min was performed. Afterward, acidification to a pH value of 3.0 was carried out by adding HCl 1N. Tetradeuterated prostaglandin F_2α_ was used as an internal standard, and two extraction steps were outperformed. Finally, the final eluate was derivatized [27]. In the mass spectrometry analysis, for 8-iso-PGF_2α_ (quantified as one of the most represented isomers of the F_2_-IsoP family), the product ion at *m/z* 299 was detected [28]. The quantitation of total F_2_-IsoPs was determined by comparison to the calibration curve (8-iso-PGF2α, Cayman Chemical, Item No. 16350, to PGF_2α_-d4, Cayman Chemical, Item No. 316010) to.

### 2.4. Resolvin D1 (RvD1) Assay

In semen samples, RvD1 amounts were measured by a quantitative sandwich enzyme-linked immunosorbent assay (ELISA) (MBS2601295-96 Rabbit resolvin D1 (RvD1) NR 1 MyBioSource). A biotin-labeled antibody and horseradish peroxidase + avidin were applied for bound biotin-labeled antibodies detection. Spectrometric detection of color intensity at 450 nm allowed the determination of semen RvD1 amounts by comparing the optical density of each seminal sample to the standard curve (ranging from 2000 pg/mL to 31.2 pg/mL RvD1 amounts).

In twenty samples (6 fertile men, 14 infertile individuals: 8 leukocytospermic and 6 varicocele infertile patients) we also made the further following investigations: clinical biochemistry determinations, analyses of sperm membrane fatty acid (FA) composition, and Phospholipase A_2_ (PLA_2_) determination. In infertile patients, these investigations were carried out when the amount of sample was sufficient.

### 2.5. Analyses of Sperm Membrane Fatty Acid (FA) Composition

From a part of the pellet, sperm membrane phospholipids were extracted and subsequently converted into fatty acid methyl esters (FAMEs) by a transesterification procedure performed in the presence of a methanol solution of 0.5 M KOH [29]. FAMEs were analyzed by a gas chromatography instrumentation (GC) (6850, Agilent Technologies, Milan, Italy) equipped with a capillary column (DB23, Agilent Technologies, Santa Chiara, CA, USA) and a flame ionization detector. Hydrogen as carrier gas and FAMEs were identified by comparison with the retention times of authentic molecules [29]. 

Palmitoleic, oleic, linoleic, eicosadienoic, eicosatrienoic, AA, EPA, docosapentaenoic (DPA), DHA acids were dosed. All the determinations were performed by the Lipidomics Laboratory at Lipinutragen Srl, CNR Area della Ricerca di Bologna, Italy.

### 2.6. Clinical Biochemistry Determinations

A semen aliquot of each ejaculate was centrifuged at 12,000× *g* to obtain a sample free of cells as reported by Feng et al. [30], and the seminal plasma was stored in 2-mL cryotubes at −80 °C until use, until thawing.

Seminal plasma samples (1 mL at room temperature) were tested using a COBAS 8000 modular analyzer (Roche Diagnostics, Mannheim, GmbH, Germany) by means of two analytical modules: C702, the high-throughput clinical chemistry module, and E602, the immunoassay module. For the analytes measured in module C702 (Fe μg/dL, CHOL mg/dL, TRSF mg/dL), COBAS 8000 calibration was done with the human lyophilized serum Calibrator C.f.a.s. (Roche Diagnostics, Mannheim, GmbH, Germany). 

Human lyophilized serum PreciControl ClinChem level 1 was used as normal control and PreciControl ClinChem level 2 was used as pathologic control (Roche Diagnostics, Mannheim, GmbH, Germany).

For the analytes measured in module E602 (FERR ng/mL, E2 pg/μL, TESTO ng/mL), we used a specific calibrator for each analyte (Roche Diagnostics, Mannheim, GmbH Germany). PreciControl Varia levels 1 and 2 (Roche 470 Diagnostics, Mannheim, GmbH, Germany), PreciControl Universal levels 1 and 2 (Roche Diagnostics, Mannheim, GmbH, Germany), and PreciControl Tumor Marker levels 1 and 2 (Roche 470 Diagnostics, Mannheim, GmbH, Germany) were used as normal and pathologic controls respectively.

### 2.7. Phospholipase A_2_ (PLA_2_) Determination

Through a process likely mediated by a PLA_2_ activity F_2_-IsoPs, which are originally formed in situ, when the FA precursor is esterified in phospholipids, are released in biological fluids in a free-form [31].

In a part of semen sample obtained after centrifugation at 400× *g* for 15 min and the pellet waste, cytosolic PLA_2_ (cPLA_2_) amounts in seminal plasma samples were determined by an enzyme-linked immunosorbent assay (ELISA, AMSBIO, kit for Human cytosolic phospholipase A_2_, cPLA_2_ (competitive), Product Code: AMS.EA 0775Hu distributed by D.B.A. Italia). In particular, the determinations were carried out as a competitive immunoassay, where standard samples (known cPLA_2_ amounts) or seminal samples were added (50 μL) to wells precoated with a monoclonal antibody to cPLA_2_. cPLA_2_ is present in the standards, or human samples competed with the biotin-conjugated antigen to bind to the capture antibody. The final revelation was performed using an avidin horseradish peroxidase. The tetrametil benzidina (TMB) substrate was then added to develop a color-changing into yellow after the addition of an acidic stop solution. Spectrometric detection of color intensity at 450 nm allowed the determination of cPLA_2_ amounts by comparing the optical density of the seminal samples to the standard curve (ranging from 16 ng/mL to 0.5 ng/mL cPLA_2_ amounts).

### 2.8. Statistical Analysis

The results were analyzed using SPSS version 23. First, the data from the study population of 85 individuals were processed. The correlation between all the relevant variables was analyzed by Spearman’s rank correlation analysis. Then, Leven’s test was performed to verify homoscedasticity. One-way non-parametric ANOVA (Kruskal–Wallis test) was used to verify the difference among groups. The Tukey Post Hoc test (or Dunnet) was performed under the significant result of ANOVA.

Second, in the group of twenty men, Mann-Whitney U was used to analyze differences between the medians of unpaired observations of two independent groups. A *p*-value of 0.05 was considered statistically significant.

## 3. Results

Semen volume, sperm concentration, progressive motility, normal morphology, and vitality were evaluated in seminal samples of 85 individuals [24]; sperm ultrastructure was also deeply investigated by TEM. The fertility index and the percentage of sperm apoptosis, necrosis, and immaturity were obtained for each sample [26]. Seminal F_2_-IsoPs and RvD1 were also dosed (Table 1). 

The correlations among the studied variables were considered using Spearman’s Rank Correlation Coefficient (Table 2).

As far as the sperm parameters are concerned, sperm concentration, progressive motility, normal morphology, and vitality showed significant positive correlations among them (*p* < 0.001). Semen volume did not reveal correlations with any considered variable.

Among TEM variables, fertility index positively correlated with all semen parameters (*p* < 0.001) and negatively with sperm necrosis and apoptosis (*p* < 0.001). Sperm apoptosis and necrosis displayed negative correlations with sperm concentration, progressive motility, normal morphology, and vitality (*p* < 0.001). Otherwise, sperm apoptosis appeared negatively associated with normal morphology (*p* < 0.01). F_2_-IsoP correlations showing a negative relationship with normal morphology (*p* < 0.05) and positive ones with necrosis (*p* < 0.05) and immaturity (*p* < 0.001). In semen samples F_2_-IsoP levels were positively correlated with RvD1 amounts (*p* < 0.001).

**Figure 1:**
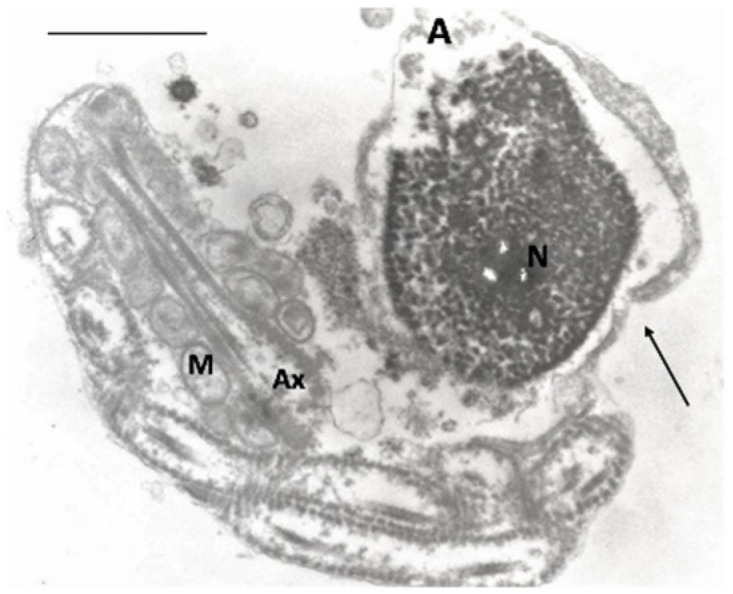
TEM micrograph of a longitudinal section of a necrotic spermatozoon with an altered nucleus and disrupted chromatin; the acrosome is empty; the axonemal components are disorganized and coiled. The plasma membrane is broken (arrow). A, acrosome; N, nucleus; M, mitochondria; Ax, axoneme. Bar: 3 µm.

**Figure 2:**
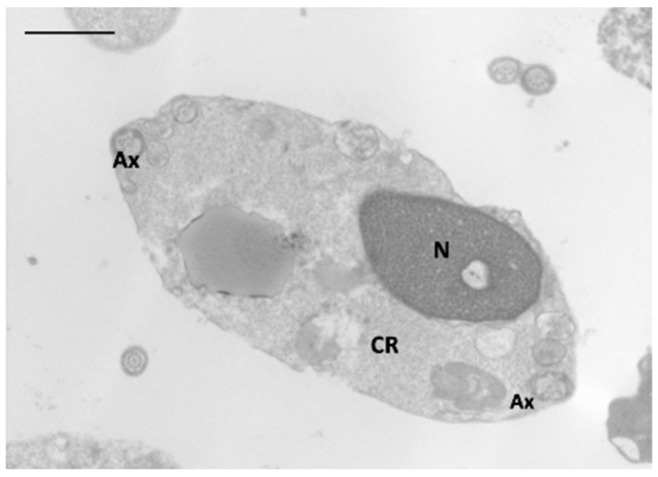
TEM micrograph of a cross-section of an immature spermatozoon with an altered nucleus and uncondensed chromatin; the acrosome is absent; the altered axoneme is coiled and embedded in a large cytoplasmic residue. N, nucleus; Ax, axoneme; CR, cytoplasmic residue. Bar: 3 µm.

RvD1 levels correlated negatively with sperm progressive motility, vitality (*p* < 0.001) and fertility index (*p* < 0.01); positively with sperm necrosis (Figure 1; *p* < 0.001) and immaturity (Figure 2; *p* < 0.01).

The selected men were divided into groups: fertile men (n° cases 18) and infertile patients (n° cases 67) according to the presence of seminal pathologies, patients with idiopathic infertility (n° cases 23), varicocele (n° cases 21) and leukocytospermia (n° cases 23, Table 3). 

In the analyzed infertile groups, sperm concentration, progressive motility, normal morphology, vitality, and fertility index were significantly decreased compared to those observed in the fertile men.

**Figure 3:**
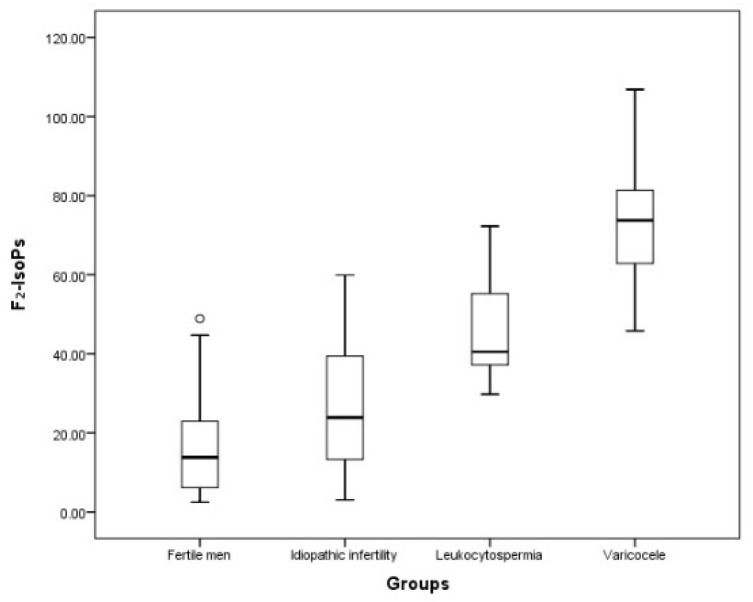
F_2_-IsoP levels in the four examined groups. Values are expressed as median and interquartile ranges (IQR). The symbol on top is an outlier.

The percentage of sperm with apoptotic characteristics was higher in the leukocytospermic (*p* < 0.001) and idiopathic (*p* < 0.01) groups than that observed in fertile men. The percentage of necrosis was significantly increased in leukocytospermic group (*p* < 0.001) compared to that detected in fertile individuals. The presence of sperm immaturity resulted significantly elevated (*p* < 0.001) in varicocele infertile patients compared to fertile, idiopathic infertility and leukocytospermic groups; this pathology significantly differed also between idiopathic patients and fertile men. Volume was not significantly different among the analyzed groups. F_2_-IsoP amount in fertile men and idiopathic infertile group resulted lower compared to leukocytospermic (*p* < 0.001; *p* < 0.01 respectively) and varicocele (*p* < 0.001, both) groups (Figure 3).

**Figure 4:**
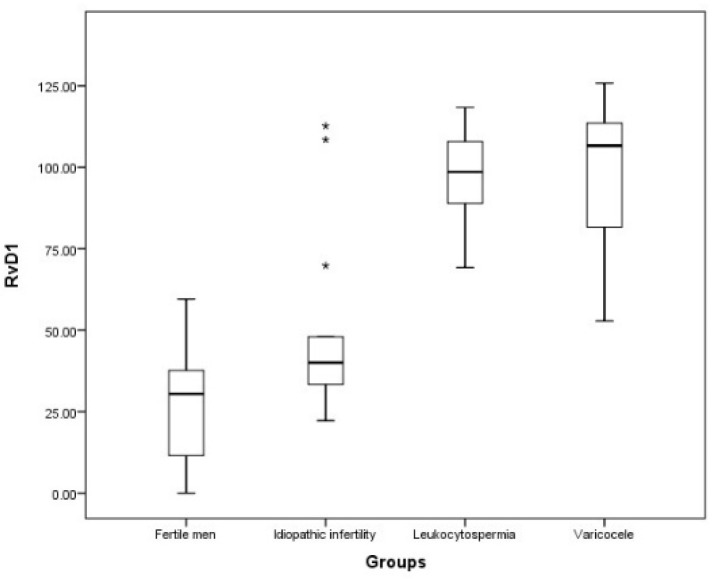
Resolvin levels in the four examined groups. Values are expressed as median and interquartile ranges (IQR). The symbols on top are the outliers.

The seminal RvD1 levels were significantly increased in patients with leukocytospermia (respectively, *p* < 0.01 and *p* < 0.001) and varicocele (both *p* < 0.001) compared to those measured in the idiopathic infertility group and fertile men (Figure 4).

The correlations among the studied variables in fertile group and each infertile group were considered using the Spearman’s Rank Correlation Coefficient. In fertile men RvD1 negatively correlated with sperm morphology (−0.521, *p* < 0.05) and F_2_-IsoPs positively with sperm immaturity (0.583, *p* < 0.05). In varicocele infertile patients RvD1 negatively correlated with sperm motility (0.465, *p* < 0.05) and in leukocytospermia positively with sperm necrosis (0.453, *p* < 0.05).

In twenty men (six fertile men, fourteen infertile patients with inflammatory pathologies: 8 with varicocele, 6 with leukocytospermia) the seminal levels of Fe, TRSF, FERR, CHOL, TESTO, and E2 were detected (Table 4). cPLA_2_ was also dosed, and the amount of different sperm FAs (palmitoleic, oleic, linoleic, eicosadienoic, eicosatrienoic, AA, EPA, DPA, DHA acids, total n-6, total n-3, EPA/AA, and n-6/n-3 ratios) were calculated (Table 4). 

All these parameters were correlated. Any correlation was observed for cPLA_2_, Fe, TRSF, and CHOL. On the contrary, RvD1 positively correlated with FERR (*p* < 0.05) and n6/n3 ratio (*p* < 0.01) and negatively with DPA, DHA, (*p* < 0.05 both), and total n-3 (*p* < 0.01). FERR had a negative correlation with DHA and total n-3 (*p* < 0.001). TESTO positively correlated with E2 (*p* < 0.01), and both negatively correlated with n6/n3 ratio (*p* < 0.01). 

The comparison between two groups: fertile men and infertile patients with inflammatory diseases highlighted significant differences (Table 5). A decrease in RvD1 and FERR levels (*p* < 0.001, *p* < 0.01, respectively) and n 6/n 3 ratio (*p* < 0.05) as well as an increase in DHA and total n-3 contents (*p* < 0.02, both) were detected in fertile group compared infertile group. 

## 4. Discussion

Resolvins are mediators as potential therapeutic agents to counteract specific chronic autoimmune and inflammatory diseases [32] and their action in the seminal fluid can be hypothesized. 

An inflammatory status may be associated with impaired quality of seminal characteristics and therefore with male infertility. Animal models have helped to understand how the presence of infection and inflammation at the testicular and epididymal levels can damage spermatogenesis [33]. 

**Figure 5:**
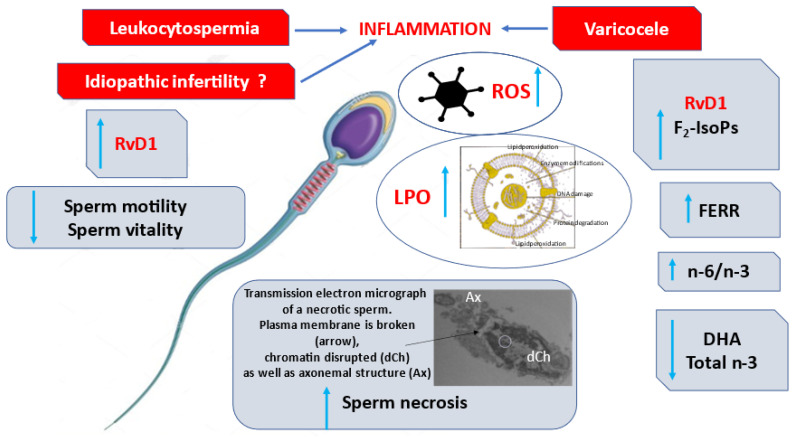
The data from the study.

This study aimed to dose the levels of RvD1 in human semen from men with different reproductive conditions (Figure 5 in relation to various other aspects such as sperm characteristics including membrane lipid composition, seminal markers, and F_2_-IsoP levels. RvD1 was detected in all analyzed human semen, and it had significant positive correlations with F_2_-IsoPs and reduced sperm quality.

As n-3 PUFAs, including DHA, are considered relevant in determining semen quality [11,34], in the present study it was intended to evaluate RvD1 as a resolvin enzymatically derived from DHA and endogenous pro-resolving and anti-inflammatory lipid mediators [35].

Lipid metabolism has been studied in inflammation-related diseases [36]; at the onset of the inflammatory response, PLA_2_ enzymes acting on membrane phospholipids mediate the release of free PUFA, including AA and the omega-3 FAs EPA and DHA. Isoprostanoids derived from AA and adrenic acid are termed isoprostanes, F_2_-IsoPs, and F_2_-dihomo-isoprostanes, respectively, otherwise, the large family of compounds derived from non-enzymatic oxidation of DHA are termed F_4_-neuroprostanes [37]. Isoprostanoid semen levels appear to be associated with male infertility being related to sperm quality and confirming the important role of FA profiling in human sperm maturation [38].

The increased RvD1 levels were associated with sperm necrosis evaluated with TEM analysis [25], a pathology characterized by disrupted chromatin. A reduction of sperm DNA integrity has been reported in presence of severe inflammation, detected in different reproductive pathologies [14]. In mice, RvD1 was associated with reduced development of cigarette smoke-induced emphysema, concurrent reductions in inflammation, oxidative stress, and cell death [39].

In our study, a relationship among inflammation, sperm necrosis, and increased amount of RvD1 that tries to counteract the inflammatory condition in seminal plasma is described. Resolvins facilitate and mediate the termination of inflammatory phases, which is an active rather than a passive process [40]. The occurrence of the chronic inflammatory process primarily depends on a persistent etiological agent. Nevertheless, chronic inflammation can also be the result of failure in inflammatory resolution when pro-resolving signaling is outweighed by pro-inflammatory stimulus [41]. Seminal inflammation may not be easily discovered and evaluation of OS or the presence of positive semen culture may not be sufficient for the diagnosis [42,43,44]. Many seminal diseases are supposed to be related to the presence of inflammatory status and identifying semen markers of inflammation is important for the diagnostic-therapeutic management of male infertility [16]. 

Therefore, we dosed RvD1 in strictly selected patients with reproductive conditions differently associated with inflammation; we decided to exclude patients with urogenital infections because of concomitant with other pathological states. Significantly, higher levels of RvD1 were assayed in the semen of patients with leukocytospermia, varicocele, or idiopathic infertility compared to fertile men. Moreover, RvD1 positively correlated with sperm necrosis in leukocytospermic patients and negatively with sperm motility in varicocele patients. 

Leukocytospermia, varicocele, and idiopathic infertility have been associated with an inflammatory status and an OS condition [19,23]. Leukocytes mediate sperm damage, including DNA fragmentation, through the secretion of ROS [45] and there is a positive correlation between leukocytospermia and IL-6 and TNF-α [46]. Varicocele is the most common abnormality being evaluated for subfertility. The pathophysiology of varicocele is not well known; three processes have been associated with the presence of varicocele: heat stress, excess of ROS, and increased apoptosis [47]. Idiopathic infertility is a peculiar condition that may be or is not linked to an inflammatory status, in this paper, the selected group of patients was probably affected by this condition. 

High RvD1 levels associated with altered sperm parameters may suggest that when chronic inflammation is present, resolvins are unable to protect male fertility, arguably because of the persistence of inflammatory stimulus(i).

Resolvin production is deficient in certain diseases which are related to chronic, unresolved inflammation, although resolvins are generated in healthy human volunteers following a dietary replenishment of DHA or EPA [48].

Recently, in subjects with chronic obstructive pulmonary disease, serum amyloid A (SAA) was identified as a biomarker for acute exacerbations [49]. SAA can also interact with ALX/FPR2 (formyl peptide receptor 2) receptors, and unlike RvD1 or lipoxin A4 (LXA4), the SAA-ALX/FPR2 interactions are pro-inflammatory [50]. Because plasma levels of SAA are at least two-log orders higher than LXA4 during acute exacerbations [51], the pro-inflammatory SAA-ALX/FPR2 signaling can overwhelm the pro-resolving mediator protective signaling at this receptor [51].

Interestingly, our data indicated that RvD1 levels increased along with other markers of OS and inflammation as FA content and clinical biomarkers. RvD1 positively correlated with FERR and n-6/n-3 ratio and negatively with DPA, DHA, and total n-3. Previously, indices of iron metabolism (FERR, Fe, and TRSF) were also associated with leukocytospermia and varicocele [23,52]. In addition, the FA composition of the sperm membrane is different between infertile and fertile men and interrelates with sperm parameters [35,53,54]. In particular, oleic acid, total MUFAs, palmitic acid, and cPLA_2_ were linked to elevated percentages of sperm necrosis, whereas DHA and total n-3 content appeared strongly positively correlated to a good sperm quality [11]. The molecular events investigated in our sperm and semen studies [11,23,52] appeared to be consistent with the condition of cell death known as ferroptosis, described as a combination of altered lipid composition, presence of oxidative stress, and excess iron [55].

We also observed that the n-6/n-3 ratio, a negative index of sperm quality, negatively correlated with TESTO and E2 and positively with RvD1. Reproductive hormones levels have been associated with the FA content and enriched diets influence testicular histology, reproductive hormones, and testicular FA profiles [56].

## 5. Conclusions

**Figure 6:**
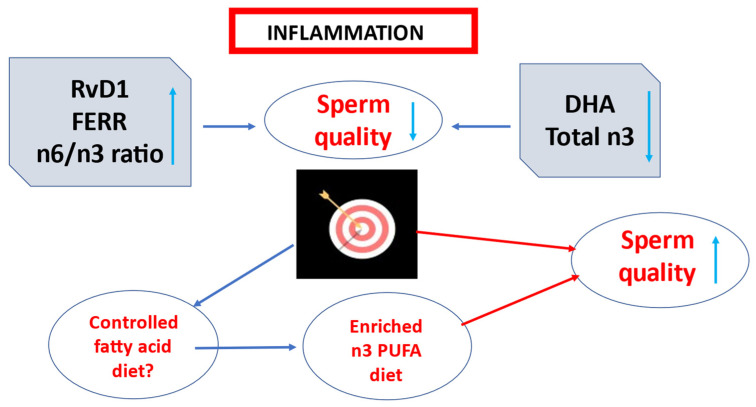
The conclusions of the study.

In this study a decrease in RvD1, FERR levels, and n-6/n-3 ratio as well as an increase in DHA and total n-3 contents detected in the fertile group strongly suggest RvD1 as indicators of a pathological seminal condition (Figure 6). The concomitant presence of high RvD1 levels associated with other seminal markers could help diagnose an inflammatory status in infertile men.

The presence of eicosanoids and resolvins are associated with the membrane amount of EPA and DHA (increase) and AA (decrease) [57]. Supplementation with specialized pro-resolving lipid mediators was an important therapeutic strategy in chronic lung disease, especially if endogenous specialized pro-resolving lipid mediator signaling was impaired [39]. Thus, enriched n-3 PUFAs diets may be useful as a treatment in male infertility involving an inflammatory status.

## Figures and Tables

**Table 1:** Medians and Interquartile ranges (IQR) of all variables in 85 individuals.

Sperm/ mL × 10^6^	Prog Mot%	NormMorp%	Vital %	FI	A%	N%	IM%	RvD1	F_2_-IsoPs
45.5 (0.3–260)	38 (0–82)	7 (1–32)	72 (10–95)	554,162.5 (2–9,732,213)	8.7 (3.4–28.3)	21.9 (12.5–76.6)	58.6 (33.2–82.4)	69.53 (4.8–125.7)	39.2 (2.5–106.8)

**Legend:** sperm/mL × 10^6^: sperm concentration, prog mot%: rapid and slow sperm motility, norm morp%: normal sperm morphology [24], vital%: live sperm with Eosin Y test, FI: fertility index [25], A: apoptosis, N: necrosis, IM: immaturity, RvD1 (pg/mL): seminal resolvins; F_2_-IsoPs (ng/mL) seminal F_2_-IsoPs.

**Table 2:** Correlations (rho Spearman’s coefficient) between all considered variables in 85 individuals.

	Sperm/mL × 10^6^	Progressive Motility%	Normal Morphology%	Vitality %	FI	A%	N%	IM%	RvD1 (pg/mL)	F_2_-IsoPs (ng/mL)
Sperm/mL × 10^6^	1									
Progressive Motility%	0.366 ***	1								
Normal Morphology%	0.647 ***	0.464 ***	1							
Vitality %	0.419 ***	0.579 ***	0.526 ***	1						
FI	0.619 ***	0.489 ***	0.716 ***	0.479 ***	1					
A%	−0.423 ***	−0.285 *	−0.435 ***	−0.408 ***	−0.522 ***	1				
N%	−0.253 *	−0.283 *	−0.302 *	−0.476 ***	−0.395 ***	0.133	1			
IM%	−0.433 ***	−0.066	−0.339 **	−0.026	−0.192	0.176	0.047	1		
RvD1 (pg/mL)	−0.136	−0.389 ***	−0.202	−0.381 ***	−0.335 **	0.163	0.558 ***	0.387 **	1	
F_2_-IsoPs (ng/mL)	−0.172	−0.075	−0.238 *	−0.133	−0.172	0.05	0.393 **	0.522 ***	0.640 ***	1

**Legend:** sperm/mL × 10^6^: sperm concentration, progressive motility%: rapid and slow sperm motility, normal morphology%: normal sperm morphology [24], vitality%: live sperm with Eosin Y test, FI: fertility index [25], A: apoptosis, N: necrosis, IM: immaturity, RvD1 (pg/mL): seminal resolvins; F_2_-IsoPs (ng/mL) seminal F_2_-IsoPs. Statistics are reported. * *p* < 0.05; ** *p* < 0.01; *** *p* < 0.001.

**Table 3:** Medians and Interquartile ranges (IQR) of all variables in the four groups (fertile men F, idiopathic infertility I, varicocele V, and leukocytospermic L). Statistics are also reported.

Variables	Fertile Men (F, n° 18)	Idiopathic Infertility (I, n° 23)	Varicocele (V, n° 21)	Leukocytospermic (L, n° 23)	Statistics Kruskal–Wallis	
					Post-Hoc Tukey	Post-Hoc Dunn
Sperm/mL × 10^6^	101.0 (56.7–127.5)	13 (1.7–52)	19 (9.5–46.2)	32 (13.6–88)	F vs. I (*p* < 0.001) F vs. V (*p* < 0.001) F vs. L (*p* < 0.01)	
Progressive motility%	52 (45.5–61)	32 (24–48)	35 (18–50)	26 (18–40)	F vs. I (*p* < 0.02) F vs. V (*p* < 0.02) F vs. L (*p* < 0.001)	
Normal Morphology%	16 (14–20)	5 (3–7)	6 (5–8.5)	8 (5–10)	F vs. I (*p* < 0.001) F vs. V (*p* < 0.001) F vs. L (*p* < 0.001)	
Vitality %	85 (79.5–90)	65 (50–72)	74.5 (70–79)	55 (50–60)	F vs. I (*p* < 0.001) F vs. V (*p* < 0.05) F vs. L (*p* < 0.001) L vs. V (*p* < 0.001)	
FI	3,218,673 (1,993,270–5,297,726)	12,234 (6811–343,253)	410,361 (218,322–835,762)	524,212 (27,261–1,131,947)	F vs. I (*p* < 0.01) F vs. V (*p* < 0.01) F vs. L (*p* < 0.05) I vs. V (*p* < 0.05)	
A%	4.7 (4.1–5.7)	11.27 (10.2–12.5)	9 (5.6–10.9)	8.7 (6.9–14)		F vs. I (*p* < 0.001) F vs. L (*p* < 0.01)
N%	23.2 (21.7–27.2)	26.7 (24–29.3)	31.7 (28–35.6)	43.3 (34–50)		F vs. L (*p* < 0.001)
IM%	53.2 (45.6–55.6)	59.9 (55.6–61.2)	75.8 (71.4–78.9)	51.6 (46.2–58.7)		F vs. I (*p* < 0.01) F vs. V (*p* < 0.001) L vs. V (*p* < 0.001) I vs. V (*p* < 0.001)
RvD1 (pg/mL)	27.9 (14.7–37.7)	42.5 (30.4–55.9)	101.1 (77.1–112.8)	97.4 (81.3–106)	F vs. I (*p* < 0.02) F vs. V (*p* < 0.001) F vs. L (*p* < 0.001) I vs. L (*p* < 0.001) I vs. V (*p* < 0.001)	
F_2_-IsoPs (ng/mL)	13.8 (6.1–23.8)	23.9 (12.3–41.2)	73.7 (62.6–82.1)	40.5 (37.2–55.8)	F vs. V (*p* < 0.001) F vs. L (*p* < 0.001) I vs. L (*p* < 0.01) I vs. V (*p* < 0.001) L vs. V (*p* < 0.001)	

**Legend**: sperm/mL × 10^6^: sperm concentration, progressive motility%: rapid and slow sperm motility, normal morphology%: normal sperm morphology [24], vitality%: live sperm with Eosin Y test, FI: fertility index [25], A: apoptosis, N: necrosis, IM: immaturity, RvD1 (pg/mL): seminal resolvins; F_2_-IsoPs (ng/mL) seminal F_2_-IsoPs.

**Table 4:** Median and Interquartile ranges (IQR) of variables in the group of 20 men (6 fertile men, 14 infertile: 8 with varicocele, 6 with leukocytospermia).

Fe	TRSF	FERR	CHOL	TESTO	E2	cPLA_2_	Palmit	Oleic	Linol	
15 (9–40)	1 (1–20)	259 (131.2–723.4)	17.5 (3–40)	1.1 (0.4–6)	51.3 (20–147.2)	0.8 (0.5–5.3)	0.5 (0.2–2)	7.2 (3–17.2)	4.8 (2.5–9.2)	
**eicosadi**	**eicosatr**	**AA**	**EPA**	**DPA**	**DHA**	**total n-6**	**total n-3**	**EPA/AA**	**n-6/n-3**	**RvD1**
0.8 (0.3–6)	0.3 (0.7–11.7)	2.55 (1–5.1)	0.2 (0.1–2.3)	1 (0.5–1.9)	19.5 (7.8–33.2)	12.1 (7–22.8)	20.8 (8.7–34.8)	0.1 (0–0.8)	0.6 (0.2–1.9)	99.8 (7.3–119.6)

**Legend:** Fe: iron (µg/dL); TRSF: transferrin (mg/dL); FERR: ferritin (ng/mL); CHOL: cholesterol (mg/dL); TESTO: testosterone (ng/mL); E2: estradiol (pg/µmL); cPLA_2_: cytosolic phospholipase; palmit: palmitoleic acid; oleic: oleic acid; linol: linoleic acid; eicosadi: eicosadienoic acid; eicosatr: eicosatrienoic acid; AA: arachidonic acid; EPA: eicosapentaenoic acid; DPA: docosapentaenoic acid; DHA: docosahexaenoic acid; total n-6: total n-6; total n-3: total n-3; EPA/AA: EPA/AA ratio; n-6/n-3: n-6/n-3 ratio; RvD1: resolvin D1, pg/mL.

**Table 5:** Median and Interquartile ranges (IQR) of RvD1, FERR, DHA, n6/n3, and total n-3 in fertile and infertile individuals. Legends: RvD1: resolvin D1 pg/mL; FERR, ferritin; DHA, docosahexaenoic acid; n-6/n-3, n-6/n-3 ratio; total n-3, total n-3. Statistics are also reported.

Patients	RvD1	FERR	DHA	n 6/n 3	Total n-3
Fertile (n° 6)	31.2 (7.3–38.1)	158.8 (131.2–246.8)	24.4 (10.1–33.2)	0.45 (0.2–0.8)	25.4 (11.3–34.8)
Infertile (n° 14)	106.0 (34.3–119.6)	283.1 (190–723.4)	16.5 (7.8–23.8)	0.6 (0.3–1.9)	17.8 (8.7–25)
Statistics	*p* < 0.001	*p* < 0.01	*p* < 0.02	*p* < 0.5	*p* < 0.02

All the other values (cPLA_2_, Fe, TRSF, CHOL, TESTO, E2, palmitoleic, oleic, linoleic, eicosadienoic, eicosatrienoic, AA, EPA, DPA, total n-6, and EPA/AA) did not show significant differences.

## Data Availability

Data is contained within the article.
